# Electrophysiological Activity and Survival Rate of Rats Nervous Tissue Cells Depends on D/H Isotopic Composition of Medium

**DOI:** 10.3390/molecules26072036

**Published:** 2021-04-02

**Authors:** Stanislav Kozin, Vladimir Skrebitsky, Rodion Kondratenko, Alexander Kravtsov, Elena Butina, Arkady Moiseev, Vadim Malyshko, Mikhail Baryshev, Anna Elkina, Stepan Dzhimak

**Affiliations:** 1 Kuban State University, 350040 Krasnodar, Russia; stas.fizika@list.ru (S.K.); aakravtsov@mail.ru (A.K.); baryshev_mg@mail.ru (M.B.); jimack@mail.ru (S.D.); 2 South Scientific Center of the Russian Academy of Sciences, 344006 Rostov-on-Don, Russia; Intro-2@rambler.ru; 3 Scientific Center of Neurology, 125367 Moscow, Russia; skrebitsky@neurology.ru (V.S.); kondrat_r@mail.ru (R.K.); 4 Kuban State Technological University, 350072 Krasnodar, Russia; butina_elena@mail.ru; 5 Kuban State Agrarian University, 350044 Krasnodar, Russia; moiseew_a@rambler.ru; 6 Kuban State Medical University of Ministry of Health of Russia, 350044 Krasnodar, Russia; 7 The V.M. Gorbatov Federal Research Center for Food Systems of the Russian Academy of Sciences, 109316 Moscow, Russia

**Keywords:** deuterium-depleted water, rats, cerebellum neurons, glucose deprivation

## Abstract

The deuterium content modification in an organism has a neuroprotective effect during the hypoxia model, affecting anxiety, memory and stress resistance. The aim of this work was to elucidate the possible mechanisms of the medium D/H composition modification on nerve cells. We studied the effect of an incubation medium with a 50 ppm deuterium content compared to a medium with 150 ppm on: (1) the activity of Wistar rats’ hippocampus CA1 field neurons, (2) the level of cultured cerebellar neuron death during glucose deprivation and temperature stress, (3) mitochondrial membrane potential (MMP) and the generation of reactive oxygen species in cultures of cerebellar neurons. The results of the analysis showed that the incubation of hippocampal sections in a medium with a 50 ppm deuterium reduced the amplitude of the pop-spike. The restoration of neuron activity was observed when sections were returned to the incubation medium with a 150 ppm deuterium content. An environment with a 50 ppm deuterium did not significantly affect the level of reactive oxygen species in neuron cultures, while MMP decreased by 16–20%. In experiments with glucose deprivation and temperature stress, the medium with 50 ppm increased the death of neurons. Thus, a short exposure of nerve cells in the medium with 50 ppm deuterium acts as an additional stressful factor, which is possibly associated with the violation of the cell energy balance. The decrease in the mitochondrial membrane potential, which is known to be associated with ATP synthesis, indicates that this effect may be associated with the cell energy imbalance. The decrease in the activity of the CA1 field hippocampal neurons may reflect reversible adaptive changes in the operation of fast-reacting ion channels.

## 1. Introduction

Changes in deuterium content can have a profound effect on the physiological processes in the body [1,2]. The creation of modern methods for deuterium-depleted water (DDW) production [3,4,5,6,7] has made possible the targeted regulation of HDO molecule levels in drinking water while tracking the response of various organs and body systems.

Available data prove that deuterium as a trace element is indispensable for normal cell function. Increasing or decreasing the concentration of deuterium in water can lead to the stimulation or suppression of various aspects of biological activity [8]. Thus, it has been shown that an increased content of deuterium in water leads to physiological, morphological and cytological changes in cells and can also have a negative impact on cellular metabolism. On the other hand, DDW, in which the content of deuterium has reduced by 30% or more, can have a beneficial effect on the organism in some conditions [9]. According to recent data, the content of isotopes in the body may change its physiological status [10].

The positive biological effects of DDW are quite diverse. Currently, the geroprotective effect of DDW in female rats [11] and its hepatoprotective effect in rats’ acetaminophen intoxication [12] has been ascertained. The positive effects of DDW consumption for a range of body protective systems were discovered [13]. In addition, the possibility of the nutrient correction of deuterium and protium distribution in the blood with the help of DDW was defined, as well as the potential benefits of the application of D/H isotope exchange reactions for the correction of the functional imbalance of nonspecific protective systems of the body [14,15].

There are also some data in relation to nervous system functioning. In particular, it was shown that DDW consumption by animals during exposure to the modeled hypoxia had a significant neuroprotective effect [16,17]. In addition, the impact of deuterium-depleted drinking for the long-term memory of rats [18] and their anxiety and stress resistance levels was noted [19,20]. These results indicate the effectiveness of DDW use as a new way of correcting a number of functional disorders [21]. This makes the study of the DDW effect on the functional parameters of an organism highly promising and relevant. However, the mechanism of these effects of DDW, in particular on the nervous system, remains largely unclear [22].

In connection with the foregoing, the purpose of this research work is to ascertain the possible mechanisms of the D/H composition modification effect on the functional activity of the nervous tissue and the survival rate of neurocytes in medium with ultralow concentrations of deuterium.

## 2. Materials and Methods

As it is known, the influence of various factors on the activity of neurons is usually researched on laboratory animals [23,24]; therefore, Wistar rats were used in our work. During the experiment, the animals were kept in standard conditions, with free access to water and food, in TECNIPLAST type IV S plastic cages, with 3–4 rats in one cage (in accordance with the rules of animal accommodation). The conditions of the animals were standardized: temperature: 20 ± 3 °C, humidity: 48 ± 2%, lighting mode: day/night (from 6.00 a.m. until 6.00 p.m./from 6.00 p.m. until 6.00 a.m.). Birch chips were used as bedding. During the whole experiment, the animals consumed standard concentrated mixed grain feed according to state standard GOST R 50258. The experiments were carried out in accordance with the requirements of the “Rules for works with experimental animals” (Order No. 708n of the Ministry of Health and Social Development of the Russian Federation dated 23 August 2010 “On approval of laboratory practice rules”), good laboratory practices (GLP), the Declaration of Helsinki (2000) and European Community Directives 86/609EEC. The study was approved by the bioethical commission of the V.M. Gorbatov Federal Research Center for Food Systems of the Russian Academy of Sciences (Protocol No. 2/2018 on 18 June 2018).

Electrophysiological studies were performed on male Wistar rats aged 5–6 weeks according to the following procedure [25]. To determine the activity of hippocampal neurons, the animals were decapitated, and 3–4 transverse sections of the hippocampus were prepared and placed into a chamber with an incubation salt solution (ISS) heated to 28 °C. In this work, two ISSs were used: they were prepared in water with different deuterium contents. ISS1 was prepared in water with natural deuterium content (150 ppm). ISS2 was prepared in deuterium-depleted water (DDW) with a concentration of 50 ppm. Both ISSs were saturated with carbogen (95% O_2_ + 5% CO_2_) and had the following composition (mM): NaCl—124; KCl—3; CaCl_2_—2.5; MgSO_4_—2.5; Na_2_HPO_4_—0.35; NaHCO_3_—26; D-glucose—10 and pH = 7.25. The flow rate was 2.5–3 mL/min. The registration of electrical activity started 2 h after the preparation of these sections (slices). The induced potential (pop-spike) in the pyramidal layer CA1 was recorded using a glass microelectrode filled with 1.5 M NaCl. The stimulation was performed with rectangular pulses with a frequency of 0.1 Hz through bipolar glass electrodes filled with a perfusing medium and placed into a radial layer of the CA1 field. The intensity of the stimulus was chosen so that the amplitude of the peak component of the response was 50% of the maximum value. The action of the medium with different content of deuterium was studied by switching the flow system from the reservoir with ISS1 to the reservoir with ISS2. After 10 min, the flow system was switched back to the reservoir with ISS1. The activity of the pyramidal cells’ population was estimated by the change in the pop-spike (PS) amplitude.

To study the tissue culture, we used 7–9-day cultures of cerebellum neurons of 8-day-old Wistar infant rats, obtained by enzymatic mechanical dissociation [26]. The cultures were grown in 96-well poly-L-lysine-coated plates in the culture medium containing 10% fetal calf serum, 2 mM glutamine, 10 mM HEPES buffer and 25 mM KCl. Twenty-four hours after the start of cultivation, we added arabinoside monocytosis (10 μM) into the cultures, used to determine the mitochondrial membrane potential and prevent the proliferation of non-neuronal cells.

To study the effect of the medium with a low deuterium content on the mitochondrial membrane potential and death rate of cerebellum neurons, two ISSs were used. One of them was prepared in water with a natural deuterium content of 150 ppm (ISS1). The other was prepared in DDW with a deuterium concentration of 50 ppm (ISS2). Both solutions had the same composition: 154 mM NaCl, 25 mM KCl, 2.3 mM CaCl_2_, 1 mM MgCl_2_, 3.6 mM NaHCO_3_, 0.35 mM Na_2_HPO_4_, 10 mM HEPES, 5.6 mM glucose. The mitochondrial membrane potential and the survival rate of the cerebellum neurons were measured on a FilterMaxF5 multifunctional reader for microtablets.

### 2.1. Intracellular Determination of Level of Reactive Oxygen Intermediate (ROI) and Measurement of the Mitochondrial Membrane Potential (MPM) of Cerebellum Neurons

To determine the level of MPM, the cultures were divided into groups according to the content of deuterium in the incubation salt solution (150 and 50 ppm) and according to the content of succinate (μM) added to the solution to activate the mitochondrial respiratory chain: 0, 25, 50, 100 and 200. To detect the MMP, tetramethyl rhodamine was used [27,28]. The probes’ loading time was 30 min at 36 °C. Then, the cultures were washed, and the fluorescence intensity was measured at an excitation wavelength of 535 nm and an emission wavelength of 595 nm. The measurement results were presented as a percentage. The fluorescence intensity of the control cultures was taken as 100% (150 ppm) without succinate.

### 2.2. The Effect of D/H Relations in the Medium on the Level of Neuronal Death during Glucose Deprivation (GD)

The cultures were divided into groups: 150 and 50 ppm without deprivation, 150 and 50 ppm + GD. Glucose deprivation was carried out for 1 h in an incubation medium of the same composition but without glucose. Then, the cultures were returned to the original salt solution with glucose and placed in a CO_2_ incubator. At 24 h, propidium iodide (PI) was added to the cultures at a concentration of 5 µg/mL for 15 min [29]. The fluorescence intensity was measured after washing the cultures at an excitation wavelength of 535 nm and an emission of 625 nm. The measurement results were presented as a percentage. The fluorescence intensity of control cultures (150 ppm) was taken as 100%.

### 2.3. The Effect of D/H Ratio in a Medium on the Level of Neuron Death Rate under Temperature Stress

The cultures were divided into groups according to the content of deuterium in the incubation salt solution (150 and 50 ppm) and according to the content of succinate (μM): 0, 25, 50, 100 and 200. Temperature stress was modeled by placing the cultures into a thermostat for 24 h at different temperatures of 22, 26, 36 and 39 °C. The survival rate was evaluated by the same method as glucose deprivation with PI addition.

The obtained data were statistically processed by methods of variation statistics using the Mann–Whitney U test to estimate the differences between independent groups and subgroups. The difference of *p* < 0.05 between groups was considered reliable. For the analysis, we used data obtained from 3 to 5 independent experiments.

## 3. Results

**Figure 1:**
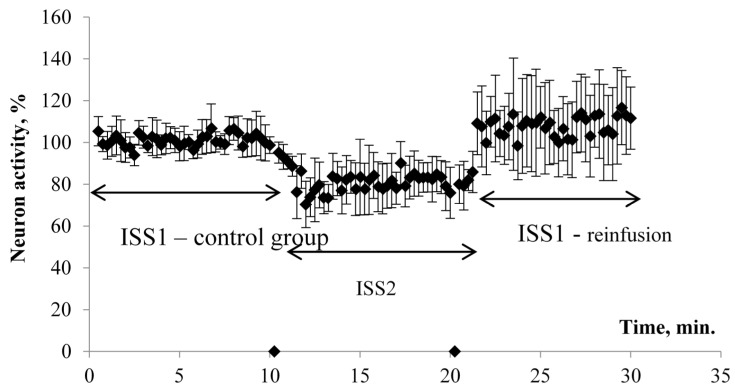
Decrease in the induced activity of neurons in the CA1 part of the hippocampus during incubation in a medium with a low deuterium content. Note: on the *Y*-axis, the pop-spike amplitude is presented as a percentage in relation to the original average value. ISS1—incubation salt solution prepared in water with a natural deuterium content (150 ppm). ISS—incubation salt solution prepared in water with a low content of deuterium (50 ppm). The arrow indicates the effect of different incubation solutions.

**Figure 2:**
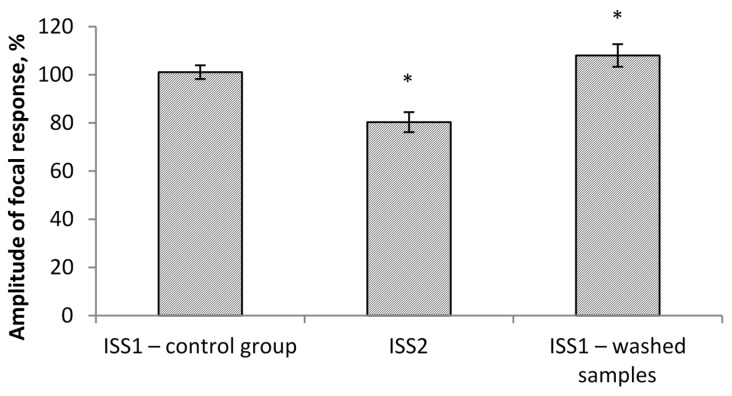
Comparison of the amplitude of focal response, expressed as a percentage in reference to the initial average of the responses (ISS1). Note: ISS1 is an incubation salt solution prepared in water with a natural deuterium content (150 ppm). ISS2 is an incubation salt solution prepared in water with a low content of deuterium (50 ppm). * *p* < 0.05 relative to ISS1—control group.

The results of electrophysiological studies are presented in Figure 1 and Figure 2. When switching the flow system and ISS1 to ISS2, the amplitude (PS) was inhibited in all the studied hippocampus sections.

The effect developed gradually and came to its maximum value on average 1.5–2 min after the switch of the flow and reached a plateau. At the same time, the PS amplitude was significantly reduced by 20% in comparison to the control group/ISS1 sample. When switching back from ISS2 to ISS1, there was a gradual restoration of the PS amplitude in all cases. The maximum effect was observed on average 1–1.5 min after the reinfusion started. The value of the restored PS was 108% of the initial value. Thus, a ten-minute incubation of the sections in the low deuterium medium led to a significant decrease of rats’ hippocampal neuron activity. The return of the slices to the incubation medium with the natural content of deuterium led not only to recovery but also to the increased hippocampal neuron population activity in comparison to the control value.

**Figure 3:**
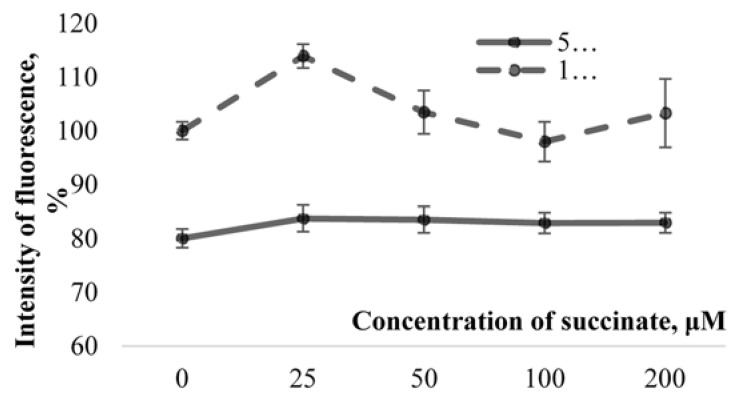
Influence of the medium with different deuterium content on the value of mitochondrial membrane potential in cultured neurons of the rat cerebellum. Note: on the Y-axis, the intensity of the fluorescence as a percentage is shown. Data are presented as M ± m as a percentage of 150 ppm at a 0 μM concentration of succinate.

The amplitude values of the pop-spike after their plateau were selected for comparison (Figure 1). The study of the effect of ISS prepared in water with a low deuterium content on the cerebellum cells’ culture showed the following results. MPM significantly decreased (*p* < 0.05) in ISS prepared in DDW at all concentrations of succinate. The differences were significant both with respect to the control (150 ppm deuterium, 0 μM succinate) and with respect to points with a similar succinate concentration in a medium with 150 ppm deuterium. The decrease in comparison to the control reached 16–20%, and between the points with equal succinate concentration, the difference was 15–30% (Figure 3).

The result analysis of the experiment to study the effect of the medium with a low deuterium content on the level of neuron death rate in GD showed the following (refer to Table 1). The rate of neuron death in GD conditions in the medium with a natural deuterium content (group “150 ppm + GD”) increased by 21 ± 3.1%, while in the medium with a low deuterium content (group “50 ppm + GD”), the death rate of neurons increased by 39 ± 4.0%, in comparison to cultures not exposed to GD in the medium with natural deuterium levels (group “150 ppm”). Moreover, in cultures placed into the medium with a low deuterium content and not exposed to GD (group “50 ppm”), the death rate of neurons was 9 ± 1.5% higher than in cultures placed into the medium with the natural level of deuterium.

Thus, GD significantly increased the death of cerebellum neurons in the medium with both natural and low deuterium levels, which is consistent with the data from the reference literature [30]. At the same time, the medium with a low content of deuterium had an additional stress effect on the culture of cerebellum neurons, which was represented by an increase in the death rate of neurons, both with and without GD.

**Figure 4:**
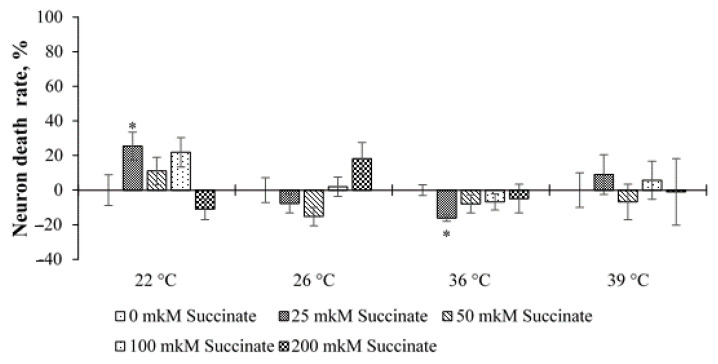
The death rate of cerebellum neurons, depending on the temperature and concentration of succinate for ISS—150 ppm. Comparison refers to the points of the 150 ppm—0 μM succinate (100%). Data are represented by M ± m. * *p* < 0.05 in relation to the point of 150 ppm. Note: 1—ISS is 150 ppm, 0 μM succinate; 2—ISS is 150 ppm, 25 μM succinate; 3—ISS is 150 ppm, 50 μM succinate; 4—ISS is 150 ppm, 100 μM succinate; 5—ISS is 150 ppm, 200 μM succinate.

The analysis of the results of the experiments with temperature stress showed that, in general, at 150 ppm deuterium, no significant effect on neuronal death was observed either in cultures with 0 µM succinate or in cultures with different concentrations of succinate (Figure 4). The only exceptions were two points at temperatures of 22 and 36 °C and 25 μM succinate. At the same time, at 22 °C, an increase in cell death was noted, and at 36 °C, a slight decrease was noted compared to the control (150 ppm, 0 μM succinate).

**Figure 5:**
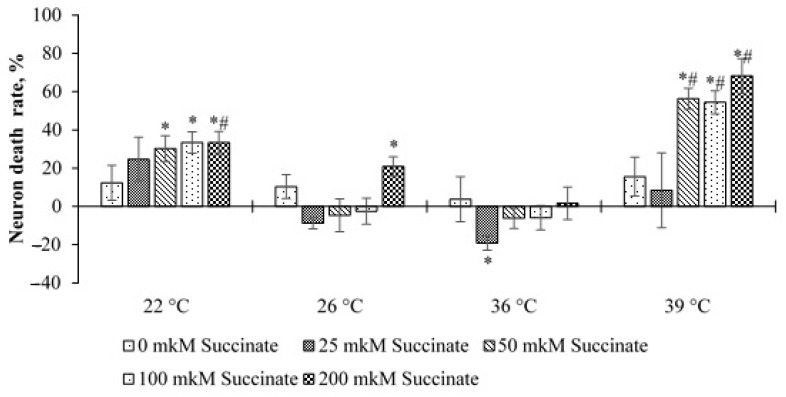
The death rate of cerebellum neurons depending on temperature and succinate concentration for ISS—50 ppm. Note: The values are compared in reference to points 150 ppm—0 μM. Data are represented by M ± m. * *p* < 0.05 in relation to the point of 150 ppm; # *p* < 0.05 is the comparison between points 150 and 50 with the same concentration of succinate. Note: 1—ISS is 50 ppm, 0 μM succinate; 2—ISS is 50 ppm, 25 μM succinate; 3—ISS is 50 ppm, 50 μM succinate; 4—ISS is 50 ppm, 100 μM succinate; 5—ISS is 50 ppm, 200 μM succinate.

A significantly different picture was observed in cultures with 50 ppm deuterium. In this case, at temperatures of 22 and 39 °C and 50–200 μM succinate, the level of cell death increased significantly compared to cultures with 150 ppm and 0 μM succinate. An increase in cell death was also noted at 26 °C and 200 μM succinate. At 36 °C and 25 μM succinate, as in cultures with 150 ppm, a decrease in cell death was noted. In addition, at 39 °C, the level of death was significantly higher in cultures placed into the medium of 50 ppm, at succinate concentrations of 50, 100 and 200 μM than that in cultures placed into the medium of 150 ppm with the corresponding concentration of succinate. At 22 °C, this was observed only for the point of 200 μM succinate (Figure 5).

These data indicate that lowering the temperature to 22 °C has a stressful effect on cells and leads to an increase in the number of dead neurons, which is more noticeable at high succinate concentrations. At the same time, at 50 ppm, this effect is more pronounced. At 39 °C, an increase in neuronal death was noted only in cultures with 50 ppm deuterium. On the one hand, this indicates a greater resistance of neurons to this temperature, and on the other hand, in the environment with 50 ppm, the stress effect is also more pronounced than at 150 ppm. This result indicates the superposition of two stress factors: a changed ambient temperature and a sharp decrease in the deuterium content. This assumption is consistent with the data obtained in experiments with GD. At the temperature of 26 °C, this effect is less pronounced, and the differences are significant only at 200 μM succinate and 50 ppm deuterium.

## 4. Discussion

Our experiments showed that the two-hour incubation of cerebellum neurons in the ISS, prepared in DDW, caused a MPM decrease at various levels of cell metabolism. The ten-minute incubation of hippocampal sections (slices) in the medium with a low deuterium content also led to a decrease in the total electrical activity of neurons. There is a connection between the two effects. According to the literature [27,28], the value of MPM correlates with the level of high-energy (macroergic) compounds produced by the cell. It is possible that the MPM decrease caused by local substitutions of deuterium for protium in cerebral tissues leads to the disruption of the synthesis of intracellular ATP. As it is known, the generation of the action potential is an energy-dependent cell process. It is likely that a decrease in ATP production in the mitochondria, as a result of the D/H-exchange in tissues, leads to changes in the Na^+^/K^+^-ATP phase. The enzyme loses its former ability to transfer sodium ions outside and potassium ions inside. As a result, the neuron loses its ability to generate an action potential.

MPM decrease, as a rule, occurs as a result of the changes in metabolic processes against the background of exposure to stress effect [31,32]. Thus, glucose deprivation, hypoglycemia and anoxia lead to a decrease in MPM in tissue culture neurons. This fact probably indicates that a 2 h incubation of cerebellum neurons in the medium with a low deuterium content has a stressful effect on the cells of tissue culture. The activation of nonspecific protective systems of the body tissues and the increase in body adaptation functions as a whole, as reported in the literature [33,34,35,36], are observed in the long exposure, which was carried out in the above-described studies.

The above-described results of the survival rate of cerebellum neurons under conditions of glucose deprivation and temperature stress confirm this hypothesis further. The death rate of cells of the nervous tissue culture with glucose deprivation is significantly higher in the medium prepared in DDW. Therefore, the percentage of the survival rate of cerebellum neurons under temperature stress also depends on the deuterium concentration in the incubation medium. The placing of cerebellum neuron cultures in ISS prepared in deuterium-depleted water leads to additional cell death with the increase of the incubation temperature to 39 °C, which is especially noticeable along with the increase of the succinate concentration. A similar situation can be observed at the low temperature of 22 °C. It can be assumed that the violation of the energy supply of neurons (the violation of ATP synthesis), caused by the substitution of deuterium for protium in the cerebral tissues, leads to the depletion of the cellular protective resources. As a result, neurons, incubated in the medium with a low deuterium content, suffered from a double-stress effect.

Moreover, perhaps, a local substitution of deuterium for protium affects some structural elements of the electron-transport chain in the mitochondria. For example, the exchange of deuterium for protium can occur in the proton channels of the inner mitochondrial membrane. In proton channels, a proton is transferred along hydrogen bonds according to the Grotthuss mechanism [37,38]. The presence of deuteron there can lead to the strengthening of hydrogen bonds and a decrease in the proton transfer rate. The substitution of deuteron for protium in the proton channel leads to a decrease in hydrogen bond energy. The weakening of the hydrogen bond leads to an increase in the potential barrier and a decrease in the proton transfer rate in the channel. The change in the kinetics of proton transfer along the channel may cause a decrease in the protons’ concentration gradient on both sides of the inner mitochondrial membrane and, as a result, lead to MPM decrease.

## 5. Conclusions

Pursuant to the conducted experiments, it can be assumed that a short exposure time of cerebrum tissue in the medium with a low deuterium content acts as an additional stress factor, thus leading to an increased neuronal death rate, especially under the terms of glucose deprivation, as well as with the combined effect of the temperature factor (22 and 39 °C) and the activation of metabolic processes against the background of the succinate concentration increase in the incubation medium to 50 µM and more. All this may be determined inter alia by the decrease in the mitochondrial membrane potential, leading to the imbalance between the functional activity and energy supply of a cell. At the same time, the decrease in the induced activity of neurons in the CA1 field of the hippocampus during incubation in the medium with a low deuterium content confirms the effect of D/H ratio changes on the functional activity of neurons, which may indicate reversible adaptive changes in the operation of fast-response (within 2 min) ion channels during the vibrations of nonradioactive hydrogen isotopes. Therefore, the use of DDW in drinking rations is advisable for the preactivation of stress-limiting systems of the body in the absence of an additional combined effect of any other stress-activating factors.

## Figures and Tables

**Table 1:** The death rate of cultured cerebellum neurons under the condition of glucose deprivation in mediums with different deuterium contents.

Group	Cerebellum Neuron Death Rate,%
150 ppm	100 ± 2.4
50 ppm	109 ± 1.5 *
150 ppm + GD	121 ± 3.1 *
50 ppm + GD	139 ± 4.0 *#

Note: Data are shown as M ± m. Here: “150 ppm” refers to cultures that were incubated in the medium with a natural deuterium content; “50 ppm” refers to cultures that were incubated in the medium with a low content of deuterium; “150 ppm + GD” refers to cultures that were incubated in the medium with a natural deuterium content and were subjected to glucose deprivation; “50 ppm + GD” refers to cultures that were incubated in the medium with a low content of deuterium and exposed to glucose deprivation (GD). * *p* < 0.05 in relation to “150 ppm”, # *p* < 0.05 in relation to “150 ppm + GD”.

## Data Availability

Not applicable.

## Sample Availability

Samples of the compounds are not available from the authors.
